# Timing of AKI after urgent percutaneous coronary intervention and clinical outcomes: a high-dimensional propensity score analysis

**DOI:** 10.1186/s12882-021-02513-9

**Published:** 2021-09-06

**Authors:** Alan S. Go, Thida C. Tan, Rishi V. Parikh, Andrew P. Ambrosy, Leonid V. Pravoverov, Sijie Zheng, Thomas K. Leong

**Affiliations:** 1 Division of Research, Kaiser Permanente Northern California, 2000 Broadway, Oakland, CA 94549 USA; 2 Department of Health Systems Science, Kaiser Permanente Bernard J. Tyson School of Medicine, Pasadena, CA USA; 3 Departments of Medicine (Nephrology), Epidemiology and Biostatistics, University of California, San Francisco, CA USA; 4 Department of Medicine (Nephrology), Stanford University, Palo Alto, CA USA; 5 Department of Cardiology, Kaiser Permanente San Francisco Medical Center, San Francisco, CA USA; 6 Department of Nephrology, Kaiser Permanente Oakland Medical Center, Oakland, CA USA; 7 Department of Medical Education, University of California, San Francisco, CA USA

**Keywords:** Acute kidney injury, Percutaneous coronary intervention, Timing, Chronic kidney disease, Death

## Abstract

**Introduction:**

Acute kidney injury is a common complication of percutaneous coronary intervention and has been associated with an increased risk of death and progressive chronic kidney disease. However, whether the timing of acute kidney injury after urgent percutaneous coronary intervention could be used to improve patient risk stratification is not known.

**Methods:**

We conducted a retrospective cohort study in adults surviving an urgent percutaneous coronary intervention between 2008 and 2013 within Kaiser Permanente Northern California, a large integrated healthcare delivery system, to evaluate the impact of acute kidney injury during hospitalization at 12 (±6), 24 (±6) and 48 (±6) hours after urgent percutaneous coronary intervention and subsequent risks of adverse outcomes within the first year after discharge. We used multivariable Cox proportional hazards models with adjustment for a high-dimensional propensity score for developing acute kidney injury after percutaneous coronary intervention to examine the associations between acute kidney injury timing and all-cause death and worsening chronic kidney disease.

**Results:**

Among 7250 eligible adults undergoing urgent percutaneous coronary intervention, 306 (4.2%) had acute kidney injury at one or more of the examined time periods after percutaneous coronary intervention. After adjustment, acute kidney injury at 12 (±6) hours was independently associated with higher risks of death (adjusted hazard ratio [aHR] 3.55, 95% confidence interval [CI] 2.19–5.75) and worsening kidney function (aHR 2.40, 95% CI:1.24–4.63). Similar results were observed for acute kidney injury at 24 (±6) hours and death (aHR 3.90, 95% CI:2.29–6.66) and worsening chronic kidney disease (aHR 4.77, 95% CI:2.46–9.23). Acute kidney injury at 48 (±6) hours was associated with excess mortality (aHR 1.97, 95% CI:1.19–3.26) but was not significantly associated with worsening kidney function (aHR 0.91, 95% CI:0.42–1.98).

**Conclusions:**

Timing of acute kidney injury after urgent percutaneous coronary intervention may be differentially associated with subsequent risk of worsening kidney function but not death.

## Introduction

Percutaneous coronary intervention (PCI) remains a first-line therapy for patients with acute coronary syndromes across the spectrum of pre-existing kidney function [1]. Acute kidney injury (AKI) is a well-recognized complication of PCI, [2, 3] with previous studies showing that AKI occurring after PCI is associated with adverse events, including incident and progressive chronic kidney disease (CKD) and death [4–8].

While there are various prior studies of the impact of AKI after PCI on future clinical outcomes with a particular focus on intravenous contrast-related management, more accurate identification of high-risk patients is needed to improve cost-effective follow-up and secondary prevention strategies, [9] especially in the current COVID-19 pandemic that has shifted a significant proportion of cardiovascular care to remote approaches [10]. At present, there is a knowledge gap in the field about when and whether to intervene peri-PCI to try to prevent AKI, as well as uncertainty about optimal preventive or management approaches related to AKI. Importantly, it remains unclear whether early detection of AKI (e.g., within 12 to 24 h) after PCI could be used to improve patient risk stratification or inform provider decisions to try to reduce the risk of subsequent AKI-related outcomes. Further complicating this is that the definition of AKI used post-PCI varies widely across studies and has not been restricted to increases in serum creatinine measurements that occur earlier than 48 h after PCI [11–13]. Additionally, the development of AKI after PCI is known to have multiple risk factors and possible mechanisms, [14–17] and it is unclear if episodes of AKI occurring at different times after PCI are related to different causes and contribute variably to different natural histories and implications for future clinical outcomes.

Our study focused on addressing the knowledge gap about whether timing of AKI in patients undergoing urgent PCI was independently and differentially associated with mortality and CKD progression after hospital discharge. We hypothesized that patients who developed AKI at different time points after PCI would experience variable subsequent risks of death and reduced kidney function than patients who do not develop AKI.

## Methods

### Source population

Kaiser Permanente Northern California (KPNC) is a large integrated health care delivery system currently providing comprehensive outpatient, emergency department and inpatient care to > 4.5 million persons in northern and central California. The KPNC membership is highly representative of the local surrounding and statewide population in terms of age, gender, race/ethnicity and socioeconomic status [18]. Nearly all aspects of care are captured through an integrated electronic health record system, with key variables extracted and standardized for research in the Kaiser Permanente Virtual Data Warehouse (VDW) [19].

This study was approved by the Kaiser Permanente Northern California institutional review board. We obtained a waiver of informed consent from the Kaiser Permanente Northern California institutional review board as the risk to patients was considered minimal given the nature of this retrospective data-only study. All methods were carried out in accordance with relevant guidelines and regulations.

### Study eligibility

We initially identified all adult (≥18 years) patients who received a PCI between January 1, 2008 and December 31, 2013 in KPNC facilities. Using previously validated methods based on electronic health records and billing claims data [20], we defined PCI using *International Classification of Diseases, Version 9* (ICD-9) or *Current Procedural Terminology* (CPT) procedure codes. Furthermore, we identified a subset of all adults receiving PCI who had detailed information on their procedure available in a regional cardiac catheterization laboratory data repository. We excluded patients who did not have an outpatient estimated glomerular filtration rate (eGFR) measurement within 12 months before the PCI, or who had Stage 5 CKD at the time of PCI based on Kidney Disease Outcomes Quality Initiative criteria [21]. We also excluded patients with less than 12 months of continuous health plan membership before their PCI or having a history of cirrhosis, organ transplantation, or renal replacement therapy. Finally, given we were interested in AKI in the high-risk setting of urgent PCI, we only included patients receiving a PCI as part of an acute inpatient or emergency department encounter for acute coronary syndrome. Each patient’s first qualifying urgent PCI during the study period was assigned as their index date.

### Acute kidney injury

Our predictor variable was AKI occurring at 12 (±6) hours, 24 (±6) hours and 48 (±6) hours post-PCI during the index hospitalization. We defined AKI as a ≥ 50% relative increase or a ≥ 0.3 mg/dL absolute increase in serum creatinine relative to the most recent pre-PCI outpatient serum creatinine measure, based on the Kidney Disease: Improving Global Outcomes (KDIGO) criteria [22]. We also characterized each instance of AKI as either Stage 1 AKI or Stage 2/3 AKI based on KDIGO serum creatinine criteria as urine output data were unavailable. We assessed AKI status for each time period independently, such that each patient was allowed to be considered to have AKI in multiple time periods. For each analysis of timing-specific AKI, we excluded any patients who did not have a serum creatinine measurement in the pre-specified time window post-PCI.

### Follow-up and outcomes

Patients were followed from hospital discharge to the first occurrence of any of the following events: death, disenrollment from the health plan, non-renal organ transplantation or end of the study follow-up period on December 31, 2014.

Our primary outcome was death from any cause, based on a previously validated comprehensive approach using electronic health records, health plan administrative data (including proxy reporting), Social Security vital status information and state death certificate data [23].

For the subgroup of patients with CKD before the PCI, we also examined post-discharge significant loss of kidney function, which we defined as a 50% relative decrease from pre-PCI eGFR using the CKD-EPI equation [24] or development of end-stage kidney disease (ESKD) defined as receipt of kidney replacement therapy based on a comprehensive health plan ESKD registry [25].

### Covariates

We identified patients with a history of proteinuria, defined as a urinary albumin/creatinine ratio ≥ 30 μg/mg, a urinary protein/creatinine ratio ≥ 0.3 mg/mg or a 24-h urinary protein measurement ≥150 mg up to 4 years before the index urgent PCI using health plan laboratory results data. We used the most recent pre-PCI serum creatinine measure to identify patients with pre-existing stage 3 or 4 CKD. We also collected all available inpatient and outpatient diagnosis codes, procedure codes and pharmacy dispensing records data up to 4 years prior to PCI for use in our high-dimensional propensity score estimation. Additionally, we identified clinically relevant comorbidities up to 4 years prior to PCI using previously validated diagnosis codes, procedure codes, laboratory and prescription medication dispensing data [26].

### Statistical approach

We used SAS statistical software, version 9.3 (Cary, NC) for all analyses, with a two-sided *P* < 0.05 being considered significant. We compared baseline characteristics across AKI status at each time point using analysis of variance for continuous variables and chi-square tests for categorical variables.

To adjust for potential unmeasured confounding, we estimated high-dimensional propensity scores (hd-PS) [27] for AKI at 12 (±6), 24 (±6) and 48 (±6) hours post-PCI, as well as for AKI at any of the three time points. To generate the hd-PS, we performed multivariable logistic regression for predicting development of AKI at each time period post-PCI using pre-PCI patient demographics, diagnoses, procedures and prescription medications, with variables selected by an algorithm that identified and prioritized candidate variables based on the empirical association between the candidate variable and the event [28, 29]. This methodology has been shown to approximate estimates of risk from randomized trials substantially better than standard propensity scoring or regression approaches [27, 30]. The final hd-PS models each included 150 algorithmically selected variables and showed excellent model discrimination for AKI at 12 (±6) hours (c-statistic 0.83), at 24 (±6) hours (c-statistic 0.90) and at 48 (±6) hours (c-statistic 0.89) post-PCI.

We conducted Cox proportional hazards models to assess the association between AKI at different time points post-PCI and each outcome of interest at 1 year of follow-up, with adjustment for pre-PCI eGFR, proteinuria status and the hd-PS for AKI at the appropriate time period. To address the possibility that AKI severity may differ across the timepoints assessed, we also conducted sensitivity analyses of the association between Stage 2/3 AKI post-PCI and all-cause mortality as well as CKD progression. We also conducted proportional hazards models for post-discharge loss of kidney function using the Fine-Gray subdistribution approach to account for death as a competing risk as an additional sensitivity analysis. Furthermore, to examine whether cardiogenic shock could account for an association between AKI and death, we conducted a sensitivity analysis excluding patients who received a diagnosis of cardiogenic shock during the index hospitalization.

## Results

### Study cohort and baseline characteristics by AKI status

**Fig. 1:**
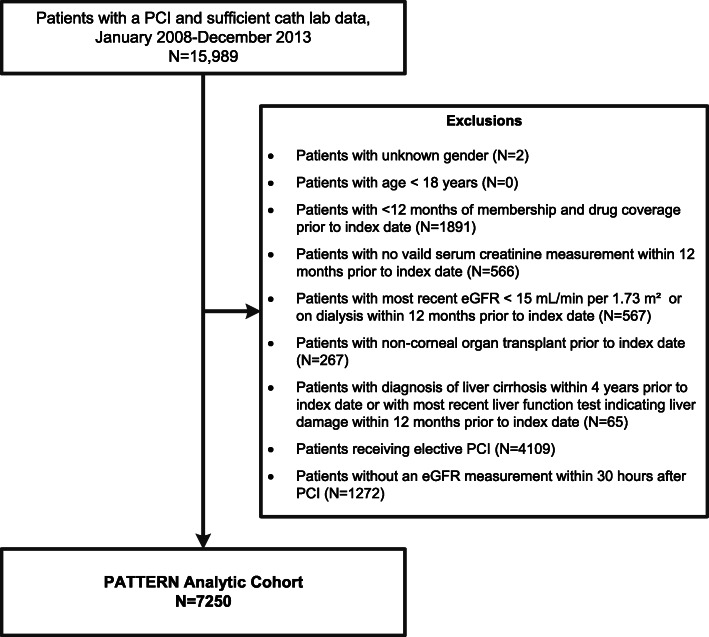
Cohort assembly diagram of adults with and without post-procedural AKI after urgent percutaneous coronary intervention identified between January 2008 through December 2013

Among the 7250 eligible patients undergoing urgent PCI identified, mean age was 67 years, 29% were women, 5% were Black and 16% were Asian/Pacific Islander (Fig. 1). Among these patients, we identified 5484 eligible patients with serum creatinine measurements at 12 (±6) hours, 2132 eligible patients with serum creatinine measurements at 24 (±6) hours, and 906 eligible patients with serum creatinine measurements at 48 (±6) hours post-PCI during hospitalization (Fig. 1). Of note, 2242 (40.9%) in the 12 (±6) hours cohort, 979 (45.9%) in the 24 (±6) hours cohort and 477 (52.6%) in the 48 (±6) hours cohort had prior Stage 3/4 CKD or proteinuria.

Across all time periods, we identified AKI in a total of 306 patients, with 152 (3%) of those with serum creatinine measured at 12 (±6) hours, 140 (7%) of those measured at 24 (±6) hours, and 147 (16%) of those measured at 48 (±6) hours. Among the identified AKI episodes, 93% of those measured at 12 (±6) hours, 85% of those measured at 24 (±6) hours and 71% of those measured at 48 (±6) hours were classified as Stage 1 AKI. At the individual patient level, 108 patients (35%) had AKI at more than one of the pre-specified time points post-PCI, with 25 (8%) having AKI at all three time points.

Patients with documented AKI at any time point were more likely than those without AKI to be older and women, and to have a lower eGFR pre-PCI (Table 1). In addition, patients with AKI were more likely to have a prior history of proteinuria, hypertension, dyslipidemia, diabetes mellitus and heart failure.

**Table 1:** Baseline characteristics of patients with and without post-procedural AKI after urgent percutaneous coronary intervention identified between January 2008 – December 2013

		48 h post-PCI		
	**Overall**	**No AKI**	**AKI**	**D-value**
	**(*N*** **= 7250)**	**(*N*** **= 6944)**	**(*N*** **= 306)**	
**Sociodemographic characteristics**				
Mean (SD) age, yr	67 (12)	67 (12)	72 (12)	0.47
Women, No. (%)	2074 (29)	1949 (28)	125 (41)	0.27
Race, No. (%)				
White	4472 (62)	4300 (62)	172 (56)	0.23
Black/African American	378 (5)	358 (5)	20 (67)	
Asian/Pacific Islander	1146 (16)	1108 (16)	38 (12)	
Other/Unknown	1254 (17)	1178 (17)	76 (25)	
Hispanic ethnicity, No. (%)	921 (13)	865 (12)	56 (18)	0.16
**Index PCI event, No. (%)**				0.24
No incident MI	1280 (18)	1248 (18)	32 (11)	
NSTEMI	4486 (62)	4283 (62)	203 (66)	
STEMI	1484 (20)	1413 (20)	71 (23)	
**Medical history, No. (%)**				
Acute myocardial infarction	501 (7)	471 (7)	30 (10)	0.11
Unstable angina	77 (1)	74 (1)	3 (1)	0.01
Coronary artery bypass graft	118 (1)	117 (1)	1 (0)	0.14
Percutaneous coronary intervention	574 (8)	553 (8)	21 (7)	0.04
Atrial fibrillation or flutter	581 (8)	543 (8)	38 (12)	0.15
Ischemic stroke/transient ischemic attack	235 (3)	218 (3)	17 (6)	0.12
Heart failure	729 (10)	665 (10)	64 (21)	0.32
Peripheral artery disease	205 (3)	180 (3)	25 (8)	0.25
Hypertension	5182 (72)	4905 (71)	277 (91)	0.52
Dyslipidemia	5520 (76)	5250 (76)	270 (88)	0.33
Diabetes mellitus	2397 (33)	2221 (32)	176 (58)	0.53
Hypothyroidism	848 (12)	794 (11)	54 (18)	0.18
Chronic lung disease	1848 (26)	1769 (26)	79 (26)	0.01
Diagnosed dementia	171 (2)	163 (2)	8 (3)	0.02
Diagnosed depression	1003 (14)	942 (14)	61 (20)	0.17
**Baseline medication, No. (%)**				
ACE inhibitor	2520 (35)	2414 (35)	106 (35)	0.00
Angiotensin II receptor blocker	1035 (14)	963 (14)	72 (24)	0.25
Beta blocker	3364 (46)	3164 (46)	200 (65)	0.41
Calcium channel blocker	1425 (20)	1315 (19)	110 (36)	0.39
Diuretic	2441 (34)	2281 (33)	160 (52)	0.40
Aldosterone receptor antagonist	99 (1)	93 (1)	6 (2)	0.05
Alpha blocker	772 (11)	712 (10)	60 (20)	0.26
Statin	3980 (55)	3781 (54)	199 (65)	0.22
Other lipid-lowering agent	478 (7)	454 (7)	24 (8)	0.05
Non-aspirin antiplatelet agent	713 (10)	657 (10)	56 (18)	0.26
Anticoagulant	384 (5)	362 (5)	22 (7)	0.08
Diabetic therapy	1390 (19)	1259 (18)	131 (43)	0.56
**Laboratory results**				
Qualifying estimated glomerular filtration rate, ml/min/1.73 m^2^, No. (%)				0.92
> 150	1 (0)	1 (0)	0 (0)	
90–150	1803 (25)	1775 (26)	28 (9)	
60–89	3571 (49)	3481 (50)	90 (29)	
45–59	1138 (16)	1069 (15)	69 (23)	
30–44	569 (8)	492 (7)	77 (25)	
15–29	168 (2)	126 (2)	42 (14)	
Documented proteinuria, No. (%)				
Yes	2036 (28)	1850 (27)	186 (61)	0.73

### Follow-up and outcomes

Mean (SD) follow-up after hospital discharge was 344 (72) days, with 403 (5.5%) observed deaths within the first year post-discharge. In addition, among 3020 patients with CKD pre-PCI, 125 (4.1%) patients experienced progressively worsening CKD during the first year of follow-up post-discharge.

### Timing of AKI and outcomes

**Fig. 2:**
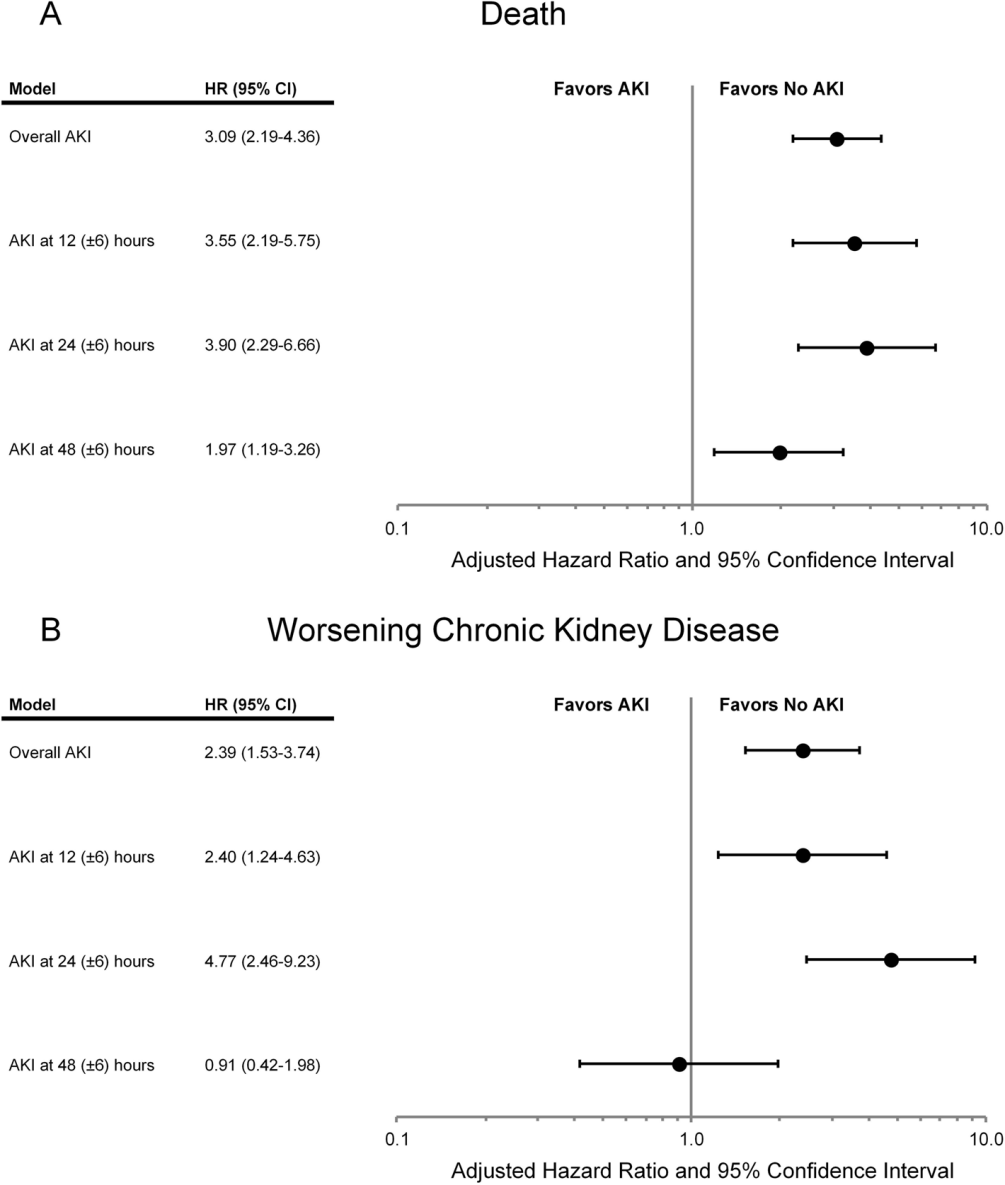
Multivariable-adjusted hazard ratios of (**A**) all-cause death and (**B**) worsening chronic kidney disease by timing of AKI after urgent percutaneous coronary intervention

In multivariable Cox proportional hazards models, compared to patients who did not experience AKI, patients who experienced AKI at any examined time point post-PCI had an increased rate of all-cause death at 1-year post-discharge, with an adjusted hazard ratio (aHR) of 3.09 (95% confidence interval [CI] 2.19–4.36). In patients with serum creatinine measurements at 12 (±6) hours post-PCI, those experiencing AKI had a more than 3.5-fold higher adjusted rate of all-cause death (aHR 3.55, 95% CI:2.19–5.75) compared to patients without AKI. Similarly, among those with serum creatinine measurements at 24 (±6) hours post-PCI, those with AKI had a nearly fourfold higher adjusted rate of death (aHR 3.90, 95% CI:2.29–6.66). In patients with serum creatinine measurements at 48 (±6) hours post-PCI, compared to those without AKI, patients experiencing AKI had a twofold higher adjusted rate of death (aHR 1.97, 95% CI:1.19–3.26) (Fig. 2A).

Among the subset of patients with CKD prior to PCI, occurrence of AKI at any examined timepoint post-PCI was independently associated with worsening CKD (aHR 2.39, 95% CI:1.53–3.74). When examining patients at specific timepoints post-PCI, occurrence of AKI at 12 (±6) hours (aHR 2.40, 95% CI:1.24–4.63) and at 24 (±6) hours (aHR 4.77, 95% CI:2.46–9.23) were both associated with significantly increased adjusted rates of worsening CKD in the first year post-discharge. In contrast, AKI detected at 48 (±6) hours, was not significantly associated with post-discharge worsening CKD (aHR 0.91, 95% CI:0.42–1.96) (Fig. 2B).

In sensitivity analyses, more severe (i.e., stage 2 or 3) AKI was associated with increased adjusted rates of all-cause death at each time point but with a declining strength of association for AKI at later time points: at 12 (±6) hours (aHR 26.53, 95% CI:8.51–82.75), at 24 (±6) hours (aHR 6.01, 95% CI:2.29–15.77) and at 48 (±6) hours (aHR 3.52, 95% CI:1.81–6.85) post-PCI. Similarly, in the subgroup of patients with CKD prior to PCI, more severe AKI was also associated with increased adjusted rates of CKD progression for severe AKI at 12 (±6) hours (aHR 104.23, 95% CI: 30.65–354.37), but not at 24 (±6) hours (aHR 2.34, 95% CI: 0.45–12.26) or at 48 (±6) hours (aHR 1.04, 95% CI: 0.36–3.01) post-PCI. Additional sensitivity analyses accounting for competing risk of death produced similar results for the associations of AKI at each time point and subsequent CKD progression.

### Sensitivity analysis of AKI timing and death in patients without diagnosed cardiogenic shock

For the subgroup of patients with no diagnosis of cardiogenic shock during the index hospitalization, in multivariable Cox proportional hazards models, compared to patients who did not experience AKI, patients who experienced AKI at any examined time point post-PCI had an increased rate of all-cause death at 1-year post-discharge (aHR 2.15, 95% CI 1.43–3.22). In patients with serum creatinine measurements at 12 (±6) hours post-PCI, those experiencing AKI had a more than twofold higher adjusted rate of all-cause death (aHR 2.39, 95% CI:1.31–4.35) compared to patients without AKI. No significant association was seen between AKI and death among those with serum creatinine measurements at 24 (±6) hours post-PCI (aHR 1.58, 95% CI:0.76–3.30) or at 48 (±6) hours post-PCI (aHR 1.32, 95% CI:0.70–2.50).

## Conclusions

Among a diverse cohort of adults undergoing urgent PCI within an integrated healthcare delivery system, we examined the associations between timing of AKI and post-discharge 1-year clinical outcomes. We observed independent associations between early AKI after PCI and all-cause mortality and in the subset of patients with pre-existing CKD, AKI was associated with CKD progression. Of note, in the subgroup of patients without a diagnosis of cardiogenic shock during the index hospitalization, the association of AKI and excess mortality was present at all time points but was only significant for early AKI. The strength and direction of the associations we observed between AKI and the outcomes of death and CKD progression are similar to previously published results for AKI identified at 48 h or later after PCI. A retrospective study of 453,475 Medicare patients undergoing PCI between 2004 and 2009 found a 2.5-fold increased rate of all-cause death within 1 year for patients with Acute Kidney Injury Network (AKIN) Stage 2/3 AKI compared to patients without AKI detected during the index hospitalization [7]. Similarly, another retrospective study of 24,405 patients in the VA Health Care system undergoing PCI between 2005 and 2010 found a twofold higher rate of all-cause death over 5 years for patients developing AKI versus patients without AKI [4]. Our study extends these previous findings by evaluating the development of AKI at three early and specific time periods post-PCI in high risk patients undergoing urgent PCI.

Specifically, AKI identified at both 12 (±6) and 24 (±6) hours post-PCI was more strongly associated with death within 1-year post-discharge than AKI identified at 48 (±6) hours post-PCI. While multiple previous analyses have consistently demonstrated an association between post-PCI AKI and excess all-cause mortality, none of the studies defined AKI based on serum creatinine results obtained less than 48 h after PCI [8]. Additionally, we found that the relative strengths of associations between AKI at different time points and all-cause death persisted when restricting the analysis to only severe AKI, despite a higher proportion of AKI at 48 h being considered severe. This suggests that the association of AKI timing with mortality may be mediated through pathways beyond just the severity of the AKI episode. We also found that AKI detected at 12 and 24 h among those with pre-existing CKD was associated with higher adjusted rates of worsening CKD post-discharge, while AKI at 48 h was not significantly associated with worsening CKD. These results suggest that AKI developing within 24 h after urgent PCI may be distinct in terms of subsequent clinical implications.

Our study has several strengths. First, we studied a relatively large cohort of 7250 patients undergoing urgent PCI who survived the initial hospitalization, which allowed us to assess the association between AKI episodes at varying times post-PCI using serum creatinine-based criteria and subsequent clinical outcomes within the 1-year post-discharge. Additionally, the comprehensive EHR data available for our cohort enabled calculation of different hd-PS’s with excellent discrimination to adjust for potential confounding at each pre-specified post-PCI time point. Our data are also more recent than other previously published studies and included a more contemporary and ethnically diverse population that support greater generalizability of the results.

Our study also had several limitations. As an observational study using serum creatinine data obtained as part of routine clinical care, not every patient received serum creatinine measures at each pre-specified time point. Thus, it is possible that the patients with serum creatinine measurements in earlier vs. later time periods post-PCI may be systematically different and also differ from patients without serum creatinine measurements, although a comparison of patients with and without measurements showed that they were similar at baseline. We limited our cohort by design to patients with urgent PCI, so our results may not completely generalize to those undergoing elective PCI in an outpatient setting, although our findings are similar to prior research on AKI occurring at any time involving multiple types of surgery and subsequent outcomes [31]. We also limited our cohort by design to patients with a serum creatinine measurement within 12 months before the index PCI procedures, so it is possible that patients without a recent serum creatinine measurement may be different than those we analyzed in this study. Additionally, we retained patients whose most recent measurement was on the index date, so it is possible that these measurements do not completely reflect the patients’ true baseline kidney function. We did not include those with Stage 5 CKD as it is difficult to identify true cases of AKI using serum creatinine-based criteria in such patients. As we did not have any urine output data post-PCI, we may have undercounted the incidence of AKI if patients did not qualify for analysis by serum creatinine criteria. We focused on all-cause mortality as a primary endpoint and CKD progression as a secondary endpoint to be most comparable with previous studies and did not address potential variable associations with cardiovascular outcomes or cardiac-related mortality, as we lacked systematic cause of death data for the cohort. We also did not have data on prehydration prior to PCI or on any IV fluids received during PCI, so were unable to determine the impact of these preventative measures on AKI and subsequent outcomes. The exact reasons for why AKI is linked to worse outcomes after PCI are not fully understood given that patients experiencing AKI may be more likely to have more extensive or complex coronary artery disease, have greater comorbidity burden, suffer hemodynamic instability, or have other characteristics that we could not fully account for in our analyses. In addition, residual kidney damage and worsening CKD after an episode of AKI can contribute to subsequent heart failure and excess all-cause mortality [32]. Our study population was derived from a large integrated healthcare delivery system in California, so our findings may not be fully generalizable to other health systems or geographic areas. Finally, we cannot completely rule out residual confounding in our models, even though we adjusted for a wide range of potential confounders using hd-PS methods, since such methods cannot adjust for unmeasured confounding that does not have a measured proxy.

In conclusion, we found that patients who develop AKI after urgent PCI experienced excess all-cause mortality at 1-year post-discharge, with a stronger association for AKI detected at 12 and 24 h than for AKI detected at 48 h. Among patients with pre-existing CKD, AKI at 12 and 24 h—but not at 48 h—after urgent PCI was also associated with higher adjusted rates of worsening CKD. Accounting for the timing of AKI development may improve risk stratification of patients undergoing urgent PCI. Further research is needed to validate if earlier vs. later AKI post-PCI has a different natural history across the range of clinical complications associated with AKI, and whether potential strategies to prevent or treat AKI vary in effectiveness based on the timing of AKI development.

## Abbreviations

AKI: acute kidney injury
CKD: chronic kidney disease
CPT: current procedural terminology
eGFR: estimated glomerular filtration rate
ICD: international classification of diseases
KDIGO: Kidney Disease: Improving Global Outcomes
KPNC: Kaiser Permanente Northern California
PCI: percutaneous coronary intervention
VDW: Virtual Data Warehouse
